# Gut mucosa alterations after kidney transplantation: a cross sectional study

**DOI:** 10.1007/s40620-024-02067-7

**Published:** 2024-09-18

**Authors:** Rashmi Joshi, Carmine Secondulfo, Alessandro Caputo, Pio Zeppa, Candida Iacuzzo, Luca Apicella, Margherita Borriello, Giancarlo Bilancio, Davide Viggiano

**Affiliations:** 1 Department of Medicine, Surgery and Dentistry, University of Salerno, Salerno, Italy; 2 Department of Translational Medical Sciences, University of Campania, Via Pansini, 5, 80131 Naples, Italy; 3 Unit of Pathology, University Hospital of Salerno, Salerno, Italy; 4 Unit of Nephrology, Dialysis and Transplantation, University Hospital of Salerno, Salerno, Italy; 5 Department of Precision Medicine, University of Campania, Naples, Italy

**Keywords:** Gut, Kidney transplant, Image analysis, Biopsy

## Abstract

**Background:**

Kidney transplant recipients (KTRs) rely on immunosuppressants like mycophenolate to prevent organ rejection. However, mycophenolate often causes intestinal symptoms and inflammation in various organs, including the skin and the colon. While KTRs have an increased risk for skin cancer, the risk of colorectal cancer is not increased.

Elucidating the histological alterations in the colon of KTRs and comparing these changes with known skin alterations would help understand how immunosuppressants influence cancer development and progression.

**Methods:**

Whole slide images from gut biopsies (Non-transplanted subjects *n* = 35, KTRs *n* = 49) were analyzed using the ImageJ and R programming environment. A total of 22,035 epithelial cells, 38,870 interstitial cells, 3465 epithelial cell mitoses, and 7477 endothelial cells, each characterized by multiple microscopy parameters, from a total of 1788 glands were analyzed. The large database was subsequently analyzed to verify the changes of inflammatory milieu in KTRs and in cancer.

**Results:**

KTRs without colon-cancer showed a significantly higher density of interstitial cells in the colon compared to non-transplanted patients. Moreover, the increase in interstitial cell number was accompanied by subtle modifications in the architecture of the colon glands, without altering the epithelial cell density. We could not identify significant structural modifications in cancer samples between KTRs and non-transplanted patients.

**Conclusions:**

Our findings demonstrate an increased number of resident interstitial cells in the colon of KTRs, as in other patients treated with mycophenolate. These changes are associated with subtle alterations in the architecture of colon glands.

**Graphical abstract:**

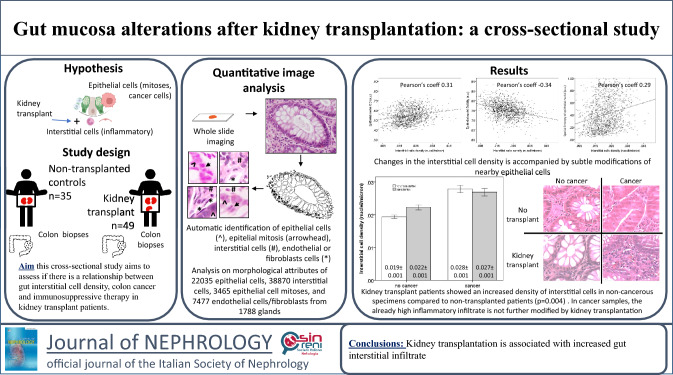

**Supplementary Information:**

The online version contains supplementary material available at 10.1007/s40620-024-02067-7.

## Introduction

Kidney transplant recipients (KTRs) frequently experience gastrointestinal symptoms, particularly indigestion and diarrhea. Studies report these symptoms affecting over half (up to 92% in some reports [1]) of KTRs, significantly impacting their quality of life [2]. Among the various immunosuppressive drugs administered to KTRs (steroids, calcineurin inhibitors, and mycophenolate), mycophenolate is suspected to be the primary culprit [2, 3]. This association is also observed in other diseases treated with mycophenolate, such as vasculitis and lupus [4]. Notably, mycophenolate is known to cause dose-dependent diarrhea [4, 5].

Histological studies suggest that mycophenolate increases epithelial cell death in both the upper [6] and lower gastrointenstinal tract [7, 8], resembling a mild form of graft-versus-host disease. These studies also reported inflammatory infiltrates associated with mycophenolate use. Similar findings were observed in experimental studies with rodents [9]. Importantly, these prior studies involved mixed samples of transplant patients, and literature data specifically conducted on KTRs are scanty. Therefore, one of our research aims is to confirm and systematically characterize increased colon inflammation in KTRs.

Low-grade gut inflammation in organ transplant patients and patients treated with mycophenolate is counterintuitive, as immunosuppressive drugs aim to suppress the immune system. However, these drugs primarily target specific components, mainly systemic T cells [10], natural killer cells [11], and neutrophils [12]. This creates an imbalance in the immune system rather than complete suppression, potentially allowing for the persistence of inflammatory infiltrates, as observed in the skin of KTRs [13–15]. Furthermore, the low-grade gut inflammation observed after mycophenolate treatment might be linked to drug-induced damage of the epithelial barrier. This barrier is very thin so as to facilitate nutrient adsorption, but also vulnerable to damage, thus necessitating a high turnover rate, with a turnover time of 1 day [16, 17]. Additionally, an innate immune system positioned right below the epithelium provides a rapid response to potential threats.

This chronic, low-grade mycophenolate-induced gut inflammation differs significantly from classic inflammatory bowel diseases like Crohn's disease and ulcerative colitis. These conditions have distinct histological features and a higher risk of colon cancer [18]. At variance, inflammatory bowel diseases, such as Crohn’s disease or ulcerative colitis, are known to increase colorectal cancer incidence, suggesting that inflammation may induce cancer development [19].Colitis patients are also at high risk of colorectal cancer [20].

In contrast, KTRs or those treated with mycophenolate [21]have little if any modification in the incidence of colon cancer compared to the healthy population, even considering some conflicting results in the literature [21–23].

Interestingly, both the colon and skin [13–15] of KTRs exhibit increased inflammatory activity (confirmed in this study with regard to the colons of KTRs). However, only the skin shows a heightened risk of non-melanoma skin cancer [13–15]in KTRs. This discrepancy is intriguing. The skin of KTRs has been reported to have a higher density of basal cells in the epithelium [13–15]. We hypothesize that, unlike the skin, the colon epithelium of KTRs will not show an increase in epithelial cell density or mitotic activity compared to non-transplanted controls.

To test this hypothesis, we provide information regarding the interstitial cells, the endothelial cells, and the overlying epithelial architecture in the colon of KTRs and non-KTRs.

## Methods

### Patients

This retrospective study was performed in accordance with the Salerno Kidney Transplant Cohort Study, reported elsewhere [24], and approved by the local Institutional Ethical Committee. The Salerno Kidney Transplant Cohort Study is an open-ended longitudinal study, based on follow-up of kidney transplant recipients self-referring to the Nephrology Unit of the Salerno University Hospital. The study included KTRs who underwent a biopsy of the lower gastrointestinal tract as per protocol or within a diagnostic path for gastrointestinal symptoms. Colon cancer screening protocols for kidney transplant patients followed the same guidelines for the general population. This included colonoscopy screening for patients over 50 years old, typically repeated every 10 years, or more frequently if a fecal occult blood test was positive. Additionally, changes in bowel habits or persistent irritable bowel symptoms (unresponsive to medication) could prompt the physician to recommend a colonoscopy on a case-by-case basis. The study included only patients with available biopsy specimens stained with hematoxylin–eosin (see below). The main exclusion criterion for transplant patients was advanced chronic kidney disease (stage IV–V) [25].

A control group was selected, consisting of non-transplanted patients who underwent biopsy of the lower gastrointestinal tract *per protocol* or within a diagnostic path for gastrointestinal symptoms; these patients were age-, creatinine- and gender-matched with KTRs.

Exclusion criteria for control subjects were administration of immunosuppressants, including the use of anti-rejection drugs, genetic predisposition to tumors, and chronic interstitial diseases. We considered the pathologic region and healthy nearby tissue for every biopsy separately. The clinical characteristics of the two groups are reported in Table 1.

**Table 1:** Baseline characteristics of the two study groups

	Non-transplanted (non-KTR)	Transplanted (KTR)	*p*
*n*	35	49	
Age (years)	60 ± 18	56 ± 12	0.2
eGFR (mL/min/1.73 m^2^)	37 ± 43	46 ± 26	0.2
Transplantation vintage	0	13 ± 7	-
Gender (male)	17 (48%)	26 (53%)	0.43
Diabetes	11 (31%)	7 (14%)	0.054
Use of calcineurin inhibitors	0 (0%)	48 (98%)	< 0.01
Use of mycophenolate	0	39 (80%)	< 0.01
Use of steroids	0	44 (90%)	< 0.01
Presence of colon cancer	9 (26%)	19 (39%)	0.155

Continuous variables are presented as mean ± standard deviation (SD). Categorical variables are presented as the number of cases (and percentage within the group). Statistical significance testing (*p*) was performed using the *t*-test for continuous variables and the Chi-square test for categorical variables

### Histological processing and whole slide imaging of intestinal biopsies

Intestinal tissues were fixed in formalin, embedded in paraffin, and cut into 5-micron thick sections. Paraffin sections mounted on slides were then deparaffinized in Xylene, rehydrated through decreasing concentrations of alcohol and then stained with hematoxylin and eosin. Slides were then dehydrated in alcohol, cleared in xylene and coverslipped in Permount. The sections were then thoroughly digitized.

Images of the biopsies were digitally acquired with a microscope (objective 20×). Several images were acquired to cover the entire region of the histological sample (in some cases up to 1 mm), taking care to have some overlap between the images. Subsequently, the entire section (whole slide images) was reconstructed using the Image Composite Editor 2.0.3.0 64bit by Microsoft. The reconstructed wide fields were then quantitatively analyzed using the ImageJ image analysis software, using the algorithm described below. Two expert pathologists performed histological diagnoses.

### Analysis of intestinal biopsies

A semi-automatic quantitative analysis system was implemented based on manually identifying the position of at least 10 intestinal glands per image masked manually, and a custom ImageJ script. Briefly, RGB images were first split into monochrome channels, and only the green channel was used for further analysis. After background subtraction (rolling algorithm) and automatic local contrast enhancement (CLAHE algorithm), a local threshold algorithm was used to identify all the nuclei in the image automatically (method Sauvola). A watershed filter was then applied to separate clustered nuclei. On these images, the “Analyze particles” command was used to identify the morphology of the gland lumen (area, centroid, shape, perimeter). Subsequently, the perimeter of the mask of each gland lumen was expanded to cover the nuclei of the epithelial cells, and the resulting ribbon covering the epithelial cells of each gland was analyzed first using the “get Profile” command and then linearized (to have a polar-like coordinate system) and each nucleus identified and measured using the “Analyze particles” command. The same method was then used to identify nuclei outside each ring of epithelial nuclei containing the interstitial peri-glandular cells.

For each nucleus, various morphometric parameters were retrieved, such as the size and position, diameter, circularity, density of nuclei per unit length of perimeter, and the number of nuclei. Similar parameters were also obtained for the surrounding nuclei to analyze the microenvironment. Therefore, an average of 50,000 variables were available for each image. To validate the method, two independent pathologists evaluated the intensity of inflammatory cells around glands using a binary scoring system: 0 (normal histology) or 1 (significant inflammation). To further characterize the epithelial layer, we measured the number of mitoses (cell division events) within the gland epithelium. We first defined morphological parameters (optical density, area, solidity, circularity, aspect ratio) of 50 mitoses and 50 non-mitotic epithelial cells identified by an expert observer. The resulting optimal parameters (solidity > 0.8, area < 15, that is small, highly compact nuclei) were then used to develop an automated identification algorithm. The algorithm's reliability was tested by comparing its results to manual counts performed by an independent observer on a larger set of images (521 mitotic events from 20 images). This comparison revealed a significant correlation between the automated and manual measurements (Pearson’s coefficient 0.51, *p* = 0.02), demonstrating the algorithm's effectiveness.

To further characterize the interstitial cell population and ensure they primarily represent inflammatory cells, we analyzed cells with elongated nuclei, indicative of fibroblasts and endothelial cells. We developed an automated method to identify these cells based on their elongated shape (circularity less than 0.2) compared to other interstitial cells. The accuracy of this method was validated by comparing automated counts to manual counts of endothelial/fibroblast cells (100 fibroblasts/endothelial cells).

**Fig. 1:**
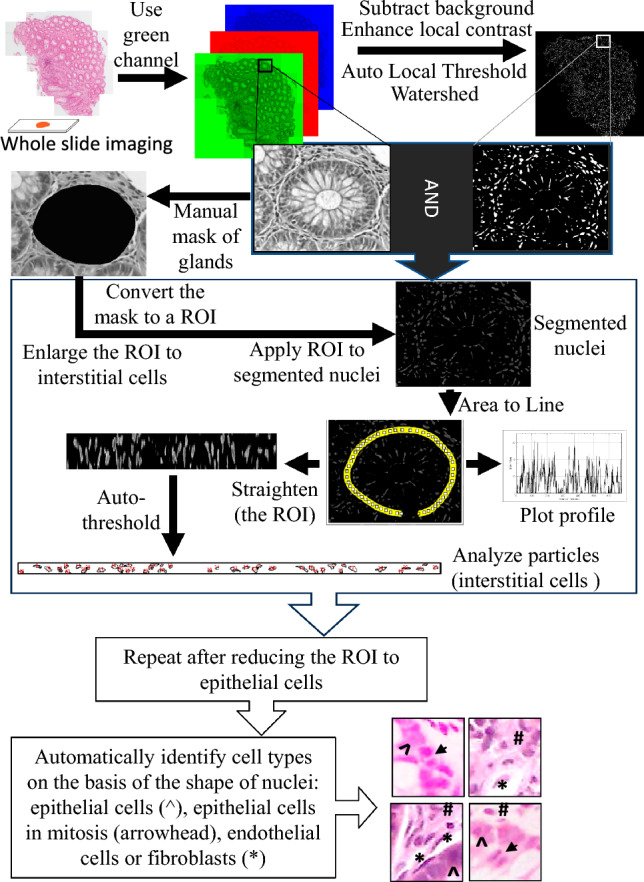
Automated image analysis pipeline for colon biopsy assessment. The figure depicts a comprehensive pipeline for analyzing images obtained from gut biopsies. The process starts with image acquisition of the entire section (whole slide imaging). Following acquisition, the pipeline progresses through several steps: (i) pre-processing (grayscale conversion, noise reduction), (ii) segmentation of nuclei to separate epithelial and interstitial cells from the background, (iii) feature extraction for each nucleus (size, shape, staining intensity, spatial distribution of gray levels), (iv) classification: based on the extracted features, algorithms can classify different cell types (epithelial cells, mitoses, endothelial/fibroblast cells, interstitial cells) and quantify their abundance in the tissue

Figure 1 shows a flow chart of the sequential steps of the methodology.

### Statistics

Whole slide imaging data allowed the analysis of each individual nucleus comprising every single gland. Since more than 1700 glands were analyzed, the total number of nuclei analyzed was greater than 60,000, each one characterized by several microscopy parameters. The resulting large dataset was analyzed using R programming environment.

Using R Programming Environment, we segregated parameters from this large dataset, such as Optical Density, area of a nuclear solidity (particle divided by its convex hull area, a measurement of the heterochromatin that is lacking optically empty regions in the nuclei), cell density (number of cells per unit length along the perimeter of the glands) in the glands or interstitial space, and spatial entropy of nuclei (a measurement of the spatial disorganization or disorder of cells). The R script to analyze the ImageJ-produced files is reported in Supplementary Material 2. The obtained data was then analyzed along with Clinical data in SPSS to study statistical significance.

To validate the semi-automatic measurement of infiltrating interstitial cells, we compared the data with a manual binary score assigned to the images by two independent observers. This binary score assessed the presence or absence of inflammation. However, only images where both observers agreed on the degree of inflammation (low or high) were included in the comparison. Images with discordant scores (observers disagreeing) were excluded to ensure reliable validation data. A logistic regression test with manually scored inflammation as the independent variable and density of nuclei in the interstitium (calculated with ImageJ) as a covariate was then used to test the predictive value of the ImageJ scores. We also analyzed the specificity and sensitivity of the automatic measure of interstitial cells for identifying inflammation using ROC curves.

Correlation of morphometric variables between interstitial cells and epithelial cells was tested using Pearson’s correlation coefficient for each gland to find correlations between morphological parameters of the epithelium and the interstitium for each gland.

Differences between the KTRs and the non-KTR control group regarding the clinical variables were tested using Student’s t-test without assuming homogeneity of variance (Welch’s method). To verify the effect of immunosuppressive therapy, we performed separate two-way ANOVAs on the following continuous dependent variables: (i) density of interstitial cells, (ii) density of epithelial cells, (iii) density of endothelial cells, (iv) density of mitoses in epithelial cells. In all cases, the use of immunosuppressive therapy (KTRs vs non-KTRs) and type of lesion (normal tissue vs cancer) were used as categorical independent factors. Continuous data are reported as mean ± standard deviation. The statistical threshold for significance was set at *p* < 0.05. The power analysis conducted using G*Power initially estimated a sample size of 84 to achieve 80% power for a medium effect size (medium effect size *f* = 0.31, rejection rate alfa = 0.05). A post-hoc analysis revealed that the actual achieved power based on our data regarding the t-tests of interstitial cells in KTRs and non-KTRs is 99%.

## Results

**Fig. 2:**
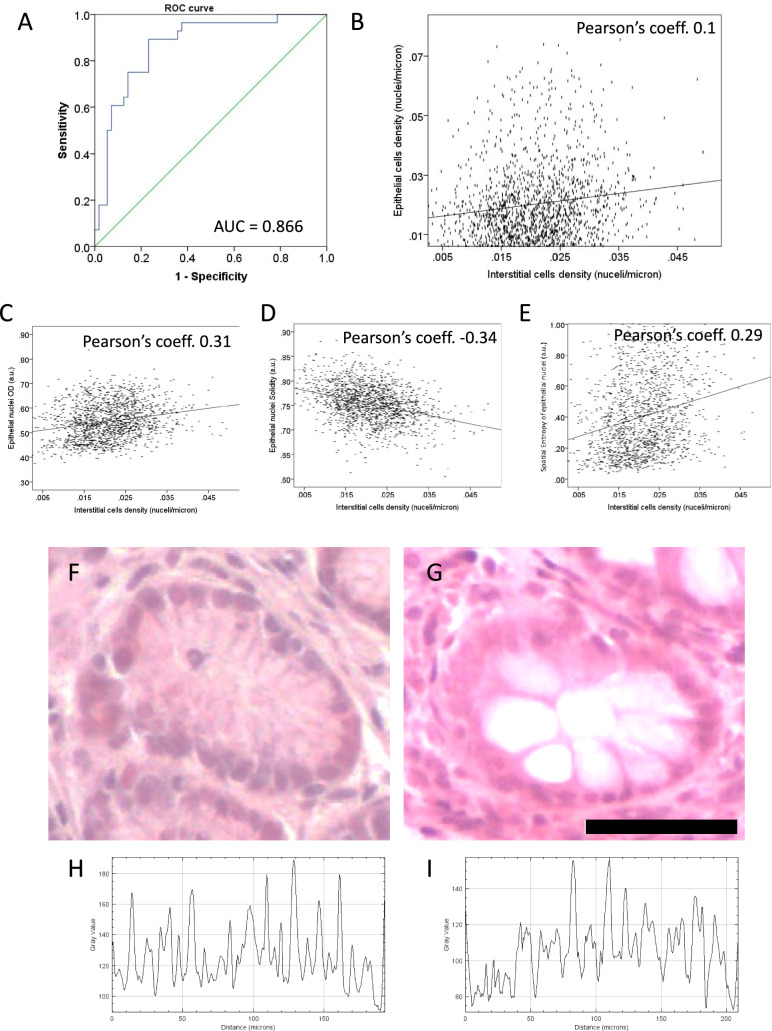
Impact of interstitial cell density on the epithelial cells. **A** Validation step of interstitial cell quantification. The Receiver Operating Characteristic (ROC curve) assesses the accuracy of the semi-automated method for counting interstitial cells. The method is evaluated for its ability to predict the presence of an inflammatory state in the tissue, as confirmed by two independent experts. **B**–**D** Epithelial cell response to interstitial inflammation. The epithelial cell density (number of cells per unit length) remains unaffected by interstitial cell density (**B**); please note that even though a statistically significant Pearson's correlation coefficient may be obtained due to the large sample size (each dot represents a gland), the magnitude of the coefficient itself is very small. This suggests a weak relationship between the variables and is not biologically meaningful. However, **C** inflammatory cells alter the staining intensity (OD: optical density) of epithelial nuclei, with higher OD indicating darker staining. Inflammation also modifies the “solidity” (a shape descriptor, panel **D**) and increases the “entropy”(measure of disorder) of epithelial cell distribution. **F**–**G** Representative images of a gland surrounded by minimal (**F**) or extensive (**G**) inflammatory infiltrate in the interstitium. **H**–**I** Quantification of the distribution of epithelial nuclei in a gland; valley depth reflects nucleus position. Lower peak-to-peak distances indicate a more clustered and potentially disrupted arrangement (higher entropy) of nuclei. Note the greater heterogeneity of peak spacing in panel I compared to panel *H*. Scale bar 50 μm

Clinical characteristics of the two study groups (transplanted vs non transplanted patients) are reported in Table 1. The morphological data derive from 1788 glands providing a total of 22,035 epithelial cells, 38,870 interstitial cells, 3465 epithelial cell mitoses, and 7477 endothelial cells, each characterized by several microscopy parameters. The semiautomatic measurement of interstitial cells used in this study was highly predictive of the presence of inflammation, manually scored by a human observer (correctly predicted cases: 81%, significance *p* < 0.001 logistic regression test). A ROC curve showed an area under the curve of 0.866 (Fig. 2A).

We then tested the effects of interstitial cells on the structure of the glands.

There was a small but significant correlation between the density of epithelial cells and interstitial cells surrounding the same gland (Fig. 2B; Pearson’s coefficient = 0.1, *p* < 0.001, *n* = 1788 glands). As shown in Fig. 2B, the correlation coefficient was very small, albeit significant due to the large number of observations, and therefore had rather limited biological meaning.

Conversely, higher interstitial cell density was associated with increased spatial entropy of epithelial cell distribution (Pearson’s coefficient 0.29, *p* < 0.01; Fig. 2E), indicating a more disordered arrangement. It also led to decreased nuclear solidity (greater euchromatin content) (Pearson’s coefficient − 0.34, *p* < 0.01; Fig. 2D) and increased hematoxylin staining intensity (Pearson’s coefficient 0.31, *p* < 0.01) of epithelial nuclei (Fig. 2C).

We then differentially compared the morphometric variables in KTRs and non-KTRs in cancer and non-cancer samples.

**Fig. 3:**
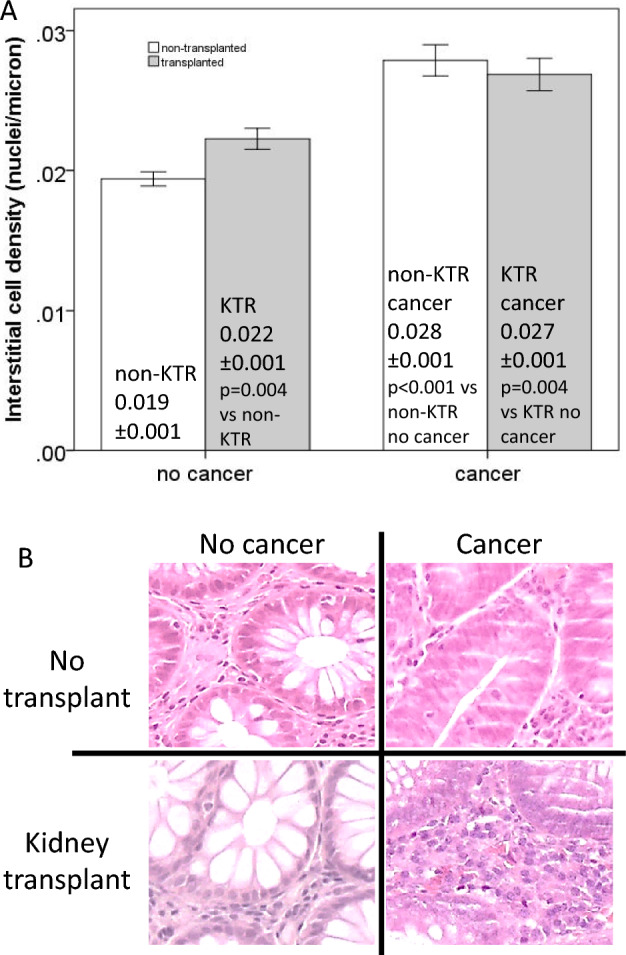
Interstitial cell density around glands quantified by the ratio of nuclei in the periglandular interstitium to the perimeter of glands (in micron). **A** Bar graph (mean ± SEM) showing the average density of interstitial cells in relation to the presence or absence of cancer and in non-transplanted (no KT) versus transplanted recipients (KTRs). **B** Magnified regions from gut biopsies, focusing on a single gland from the four groups i.e. (i) no cancer and no transplant, (ii) cancer and no transplant, (iii) no cancer and kidney transplant, (iv) cancer and kidney transplant

Kidney transplantation significantly increased the density of interstitial cells in non-cancerous tissue samples by 15% (Interstitial cell density: non-KTRs 0.019 ± 0.001 cells/micron; KTRs 0.022 ± 0.001cells/micron; *p* = 0.004 *t*-test). This effect was not observed in cancerous tissues (Interstitial cell density: non-KTRs 0.028 ± 0.001 cells/micron; KTRs 0.27 ± 0.001 cells/micron; *p* = 0.5 *t*-test). Indeed, a two-way ANOVA of interstitial cell density using transplant (two levels) and cancer (two levels) as factors confirmed that the increase in interstitial cells was specific to non-cancerous tissue and dependent on the presence of a transplant (*F* = 1.21, *p* = 0.043 for interaction effect); furthermore, the presence of cancer was also accompanied by increased inflammatory infiltrate (cancer factor *F* = 47, *p* < 0.01) (Fig. 3).

Further analysis revealed no significant changes in endothelial cells/fibroblasts or mitotic epithelial cells due to kidney transplantation (endothelial cells: KTR factor *F* = 0.53, *p* = 0.47; mitoses: KTR factor *F* = 1.54, *p* = 0.218), although both cell types were more abundant in cancerous tissues (cancer factor, endothelium *F* = 6.5 *p* = 0.013, mitoses *F* = 9.0, *p* = 0.004, respectively), without significant interaction effect.

## Discussion

Our study identified distinct alterations in the gut structure of KTRs compared to non-transplanted controls: (i) increased number of interstitial cells in non-cancerous tissue of KTRs: these patients exhibited a significant increase in the number of interstitial cells within the colon, suggesting a heightened inflammatory response. This finding is further supported by the strong agreement between manual observations of inflammation and automated quantification of interstitial cells; (ii) no change in epithelial or endothelial/fibroblast cell density in KTRs: importantly, we observed no significant changes in the density of epithelial cells, endothelial cells/fibroblasts, or epithelial cell division rates (mitosis) in KTRs compared to controls. Only subtle changes in the epithelium (Optical Density of the nuclei, spatial entropy, solidity) were correlated with the extent of inflammatory infiltrate. This suggests the proliferation of the colonic epithelium remains unaffected by immunosuppressive therapy; (iii) increased mitosis and epithelial cell density in cancerous tissue: across all patient groups (KTRs and non-KTRs), cancerous colon tissue displayed a higher density of epithelial mitoses, endothelial cells, and interstitial cells compared to healthy tissue. This observation aligns with the expected characteristics of cancerous growth.

Our findings support the initial assumption that immunosuppressive therapy administered to KTRs may enhance inflammatory activity in the colon. This could be partially mediated by mycophenolate, a common immunosuppressant, as supported by existing literature on its pro-inflammatory effects in the gut [7]. This aligns with the observed higher inflammation in the colon of other solid organ transplant patients and those treated with mycophenolate [6, 26].

The impact of KT on cell proliferation appears to be tissue-specific; the lack of observed increased cellular density in the colon epithelium aligns with epidemiological data indicating no association between treatment in KTRs and colon cancer risk [21, 27]. A small increase in colorectal cancer has been reported in the case of heart transplants [28]. Conversely, the known risk of skin cancer in these patients might reflect the observed increase in basal cell density in the skin of KTRs [15].

One speculation we advance for future work is based on the observation that the protein target of calcineurin inhibitors (used in KTRs), that is Peptidylprolyl isomerase A (the binding protein of Tacrolimus) is expressed in the keratinocytes but not in enterocytes (data from the human protein atlas), whereas the target of mycophenolate is present in both cell types. Therefore, it is tempting to attribute the increased inflammation to mycophenolate and the cancerogenic effect to calcineurin inibitors [23]. Indeed, FK506 (tacrolimus) and cyclosporin, two calcineurin inhibitors used in kidney transplants, have been reported to impair intestinal permeability in a dose-dependent manner in animals [29] and in humans [30]. However, this effect has been downsized more recently [Supp 31].While this hypothesis offers a potential explanation for the differential cancer risk in skin versus colon in KTRs, further research is necessary to validate it, as well as to investigate the role of Peptidylprolyl isomerase A in cancer development.

Our study has some limitations. As a single-center retrospective study, the generalizability of the findings to other patient populations needs further investigation. Additionally, the retrospective design does not allow us to definitively establish a cause-and-effect relationship between KTRs and the observed changes in interstitial cell density.

However, several aspects suggest a potential link between KTRs, immunosuppression, and colon inflammation:

1. skin Inflammation parallels colon inflammation in KTRs: a different cohort of KTRs compared to controls showed skin inflammation [15], supporting the notion that immunosuppression might contribute to low-grade inflammation.
2. mycophenolate's inflammatory effects: mycophenolate, a common immunosuppressant, is known to cause dose-dependent inflammatory reactions in the gut, resembling mild graft-versus-host disease [6].
3. gut inflammation in other mycophenolate users: studies report gut inflammation in patients receiving mycophenolate for conditions like vasculitis [4].
4. high prevalence of gastrointestinal symptoms in KTRs: a large percentage of KTRs experience gastrointestinal symptoms, potentially linked to our observed gut inflammation data [5, Supp 32].

Another limitation is that we could not definitively identify the type of interstitial cells using immunohistochemistry. However, the analysis of endothelial/fibroblast cells suggests that the increased number of interstitial cells is very likely represented by inflammatory cells rather than, for example, fibrosis or angiogenesis. As suggested by a reviewer, we used the broader term “interstitial cell” throughout the manuscript.

Despite these limitations, our study offers a valuable contribution. The lack of data on the structural gut alterations in KTRs makes this work significant for the nephrology community and is likely to inspire further research. Furthermore, considering the high prevalence of gut discomfort associated with mycophenolate and the critical importance of improving patient quality of life, our histopathological findings offer valuable insights.

## Conclusion

Kidney transplantation was related to increased interstitial cell density in cancerous and non-cancerous tissues. These interstitial cells are correlated to subtle modifications of the architecture of the epithelial layer in colon glands. Due to the limited existing data on gut alterations in KTRs, this study represents a significant first step in the understanding of gut symptoms in KTRs and the different occurrence of cancer in skin vs colon in KTRs, despite similar inflammatory reaction. Our findings have the potential to pave the way for improved treatments and preventative measures to address the main gastrointestinal side effects associated with immunosuppressive treatments.

## Supplementary Information

Below is the link to the electronic supplementary material.

Supplementary file1 (DOCX 19 KB)

## Data Availability

The datasets generated and/or analyzed during the current study are available from the corresponding author (DV) on reasonable request.
