# Supplementary material for: Gut mucosa alterations after kidney transplantation: a cross sectional study
Source: J Nephrol. 2024 Sep 18;38(4):1153–62. doi: 10.1007/s40620-024-02067-7 (PMC12187894; doi:10.1007/s40620-024-02067-7)
Supplement: Supplementary file 1 — Supplementary file1 (DOCX 19 KB) [file 40620_2024_2067_MOESM1_ESM.docx]

**Supplementary material**

**References not included in the main test:**

Supp 31. Parrilli G, Abazia C, Sarnelli G, et al (2003) Effect of chronic administration of tacrolimus and cyclosporine on human gastrointestinal permeability. Liver Transpl 9:484–8. https://doi.org/10.1053/jlts.2003.50088

Supp 32. Arns W (2007) Noninfectious gastrointestinal (GI) complications of mycophenolic acid therapy: a consequence of local GI toxicity? Transplant Proc 39:88–93. https://doi.org/10.1016/j.transproceed.2006.10.189

ImageJ macro for automatic epithelial cell quantification from WSI

Note: a mask with the inner part of the glands must have been created manually or using other semiautomatic methods.

// Automatic identification of gland cells and their morphological characteristics. By Davide Viggiano 2022

// a 8bit RGB image of gut histology should be available together with the same image with gland lumens painted in black (mask)

// every gland must become a ROI for every gland we retrieve the size and position, and diameter, the number of nuclei, the density of nuclei per unit length of perimeter

// the sequence of nuclei and their staining intensity, the homogeneity of the chromatin, the statistics of their distribution

// we also want similar statistics immediately on the border of the gland to analyze the microenvironment

// delete all previous ROIs

n=roiManager("count");

if (n>0) {

roiManager("Delete");

}

// load the image

img = File.openDialog("Select starting image");

folder = File.getDirectory(img);

pz = File.nameWithoutExtension ;

open(img);

// saet the scale; this should be changed according to the resolution of the images

run("Set Scale...", "distance=10.7703 known=7 unit=microns global");

run("Split Channels");

blue = getImageID(); // get rid of blue image

close();

red = blue+2 ; //assign ids

selectImage(red);

close();

imgID = getImageID(); //assign ids

setMetadata("Label", "imgID starting image");

// now open the masks

selectImage(imgID);

run("Subtract Background...", "rolling=200 light");

run("Enhance Local Contrast (CLAHE)", "blocksize=9 histogram=256 maximum=3 mask=*None* fast_(less_accurate)");

run("Duplicate...", " ");

masknucleiID= getImageID(); //masknuclei is the image thresholded for nuclei

setMetadata("Label", "masknucleiID nuclei thresholded");

run("Auto Local Threshold", "method=Sauvola radius=15 parameter_1=0 parameter_2=0");

run("Invert");

run("Watershed");

run("Invert");

imageCalculator("AND create", imgID,masknucleiID); // ora dell’immagine di partenza abbiamo solo I nuclei

onlynucleiID= getImageID();

setMetadata("Label", "onlynucleiID");

//this one contains the profiles of nuclei, with their initial grayscale Values;

// variables: imgID=initial image;folder=path; pz=filename

// now let us convert the gland masks into ROIs

mask = File.openDialog("Select MASKS image");

open(mask);

maskID= getImageID();

setMetadata("Label", "maskID");

selectImage(maskID);

setThreshold(0, 8);

setOption("BlackBackground", false);

run("Convert to Mask");

run("Clear Results");

run("Set Measurements...", "area mean standard centroid shape integrated limit redirect=None decimal=3");

run("Analyze Particles...", "size=10-Infinity display exclude clear include add");

// save the position of the glands...

saveAs("Results", folder+pz+"glands positions.csv");

// and now let us start the analysis!!

selectImage(onlynucleiID);

n = roiManager("count"); //iterate on each ROI i

for (i=0; i<n; i++) {

selectImage(onlynucleiID);

roiManager("select", i);

run("Enlarge...", "enlarge=6"); //we assume that the border of the ROI is outside the nuclei. For our images, a border 15pixels apart from the existing one will pick up the periglandular cells

run("Line Width...", "line=18");

run("Area to Line");

profile = getProfile();

// now place the profile in the result table

Table.create("microenvironment profile")

Table.setColumn("gray value", profile);

Table.save(folder+pz+i+"microenvironment profile.csv")

run("Close");

selectImage(onlynucleiID);

run("Straighten..."); //first linearize the nuclei so that the coordinates are on a polar coordinate

streightenedID= getImageID();

setMetadata("Label", "streightenedID");

setAutoThreshold("Default dark");

run("Analyze Particles...", "size=6-100 display clear include");

// this will analize nuclei as particles; it is different from the grayscale profile above

saveAs("Results", folder+pz+i+"microenvironment particles.csv");

run("Clear Results");

selectImage(onlynucleiID);

roiManager("Select", i);

run("Enlarge...", "enlarge=-6"); // we assume that the border of the ROI is outside the nuclei. For our images, a border 15pixels apart from the existing one will pick up the periglandular cells

run("Line Width...", "line=18");

run("Area to Line");

profile_i = getProfile();

// now place the profile in the result table

Table.create("gland profile")

Table.setColumn("gray value", profile_i);

Table.save(folder+pz+i+"gland profile.csv")

run("Close");

selectImage(onlynucleiID);

run("Straighten..."); //first linearize the nuclei so that the coordinates are on a polar coordinate

streightenedIDi= getImageID();

setMetadata("Label", "streightenedIDi");

setAutoThreshold("Default dark");

run("Analyze Particles...", "size=6-100 display clear include");

saveAs("Results", folder+pz+i+"gland particles.csv");

run("Clear Results");

}

close("*");

selectWindow("Results");

run("Close" );

run("Close All" );

// in the resulting files the number of glands, number of cells, length of glands are also available for further analysis

Supplementary material 2

R script for the analysis of files produced by ImageJ macro

# This script reads all the files created by the ImageJ macro and stored in a single folder, importing all the data into a single

# dataframe “results” which can be saved for further analysis,

results <- data.frame (name='x',gland='x',inorout='x', n=1,n_area=1, OD=1,circ=1,length_p=1)

# name: subject name , gland= gland number within the image, n = nuber of nuclei; this must be divided by length of the

# profileto get thenuclear density= n_density

# OD : mean optical density of nuclei, circ_i: mean circularity, n_area= mean size of nuclei inside,

# length_p = length of the profile (or of the gland) ,inorout 0= outside = interstitial cells; 1= inside= epithelial cells

# int: interstitial cells = n_int, n_area_int, OD_int, circ_int

# ins: inside=epithelial cells:ins_pro, int_pro, n_density_ins,n_density_int

directory <- choose.dir(default = "", caption = "Select folder")

filename = list.files (directory)

setwd(directory)

for ( file_n in c(1:length(filename) ) ) {

curr_file<- filename [file_n]

gland <- substring(sub(".*stitch", "", curr_file)[[1]],1,1)

name <- sub("stitch.*", "", curr_file)

curr_row<- nrow(results) + 1

results[curr_row,] <- NA

if ( grepl("profile", curr_file, fixed = TRUE) ) {

inorout<- "profile"

profiles = read.csv(curr_file)

#FIRST CALCULATE THE optic density OD

profiles <- -log10(profiles/255)

names(profiles) <- "OD"

length_p<- nrow(profiles)

results$length_p[curr_row] <- length_p

results$inorout[curr_row] <- inorout

}

if ( grepl("particles", curr_file, fixed = TRUE) ) {

inorout<- "particles"

particles = read.csv(curr_file)

#FIRST CALCULATE THE optic density OD

particles$OD<- -log10(particles$Mean/255)

results$n<- nrow (particles)

results$n_area<- particles$Area

results$OD<- particles$OD

results$circ<- particles$Round

results$inorout[curr_row] <- inorout

}

results$name<- name

results$gland<- gland

}
